# ASO Author Reflections: Lost in Transition: Addressing Preventable Attrition after Neoadjuvant Chemotherapy

**DOI:** 10.1245/s10434-025-17887-5

**Published:** 2025-07-20

**Authors:** Jaspinder Sanghera, Michelle Holland, Ioannis Liapis, Annabelle L. Fonseca

**Affiliations:** 1 Department of Surgery, The University of Alabama at Birmingham, Birmingham, AL USA; 2 Institute for Cancer Outcomes and Survivorship, The University of Alabama at Birmingham, Birmingham, AL USA

## Past

The integration of neoadjuvant chemotherapy into the treatment paradigm for nonmetastatic pancreatic and gastric cancer has become increasingly common, reflecting evidence that supports its potential to improve oncologic outcomes through early treatment of micro-metastatic disease, improvement in surgical selection, and tumor downstaging.^1,2^ However, previous research has demonstrated that up to 15–38% of patients fail to reach curative-intent resection following completion of therapy.^1,3^ While disease progression accounts for some of these cases, other nontumor biology-related causes of attrition remain underexplored. Understanding and addressing the modifiable causes of attrition represents a critical opportunity to improve the delivery of guideline-concordant treatment (GCT). This study aimed to identify and characterize the mechanisms leading to modifiable attrition after neoadjuvant chemotherapy, and to evaluate the association of patient- and system-level factors with treatment completion in a vulnerable population at a safety-net and tertiary care institution in the Southeastern USA.^4^

## Present

In this retrospective single-institution study, we evaluated patients with potentially resectable pancreatic or gastric cancer who received neoadjuvant chemotherapy during the study period. A detailed root cause analysis was performed to delineate the underlying factors contributing to attrition. Multivariate logistic regression was used to examine the association between sociodemographic and access-related factors with attrition. Of those who initiated neoadjuvant chemotherapy, 49% failed to proceed to surgery for reasons unrelated to tumor progression or anatomic unresectability. This group of patients experienced worse overall survival, regardless of cancer type. Older age, diagnosis of pancreatic cancer, and three or more emergency department (ED) visits after diagnosis were significantly associated with increased likelihood of attrition.

On root cause analysis, we identified four primary drivers of nontumor biology-related attrition: (1) physical deconditioning, due to treatment toxicity, malignancy or procedural complications; (2) loss to follow-up resulting from missed appointments and lack of system-initiated monitoring; (3) healthcare delivery deficiencies including delayed or absent referral to specialists; and (4) patient refusal of treatment due to competing responsibilities or patient beliefs. Potential strategies to mitigate modifiable attrition include implementation of structured pre-habilitation programs to improve or maintain functional status during treatment, oncology symptom management clinics to proactively address treatment-related toxicities, and establishment of cancer care navigation services to facilitate care coordination, prevent or re-engage patients lost to follow-up, and address logistical or socioeconomic barriers.^5^ Facilitating patient access to primary care providers will not only potentially allow for more rapid initiation of diagnostic workup, but also could reduce preventable ED admissions. Improving inter-provider communication, standardizing referral pathways to surgical oncology, and embedding surgical oncology consultation and planning earlier in the treatment timeline may help ensure continuity of treatment.

## Future

This study underscores the considerable prevalence of potentially preventable attrition following neoadjuvant chemotherapy and represents the first step toward improving the understanding of underlying contributing factors while highlighting potential future solutions. As neoadjuvant chemotherapy becomes increasingly utilized in the management of foregut malignancies, it is important to focus our attention not only on treatment efficacy, but also on implementation science and healthcare delivery optimization, particularly to safeguard access to guideline-concordant treatment among medically and socioeconomically vulnerable patient populations. This study was conducted at a safety-net hospital in the southeastern USA, an area characterized by poor health outcomes and high levels of poverty, and as such, these results may not be universally generalizable. Future studies should aim to validate these findings in broader institutional and geographic contexts. Granular, institution-level data are essential to identify context-specific systems-based gaps and to inform targeted interventions, particularly within resource-constrained environments. Qualitative research to explore patient perspectives and lived experiences is also vital to reveal the often unseen social, logistical, and other barriers to completing neoadjuvant chemotherapy and proceeding to surgery. The development of prediction models integrating clinical and sociodemographic data, together with qualitative data, could allow for the early identification of patients at high risk of attrition, thereby guiding preemptive supportive interventions. Ultimately, reducing nontumor biology-related attrition during neoadjuvant chemotherapy requires deliberate, system-wide commitment. Efforts to reduce attrition will improve our clinical outcomes while also reflecting our responsibility to design healthcare pathways that are responsive, inclusive, and attuned to the needs of the patients we serve.
